# Factors Determining Work Arduousness Levels among Nurses: Using the Example of Surgical, Medical Treatment, and Emergency Wards

**DOI:** 10.1155/2019/6303474

**Published:** 2019-12-31

**Authors:** Krystyna Kowalczuk, Elżbieta Krajewska-Kułak, Marek Sobolewski

**Affiliations:** ^1^Department of Integrated Medical Care, Medical University of Bialystok, Bialystok, Poland; ^2^Faculty of Management, Rzeszow University of Technology, Rzeszow, Poland

## Abstract

**Introduction:**

Staff shortages among nurses have been severely felt in most countries around the world for many years. In Poland, this problem is particularly visible due to the lowest nursing employment rate per 1000 inhabitants among 28 EU states and the high rate of leaving the profession. The average age of Polish nurses has been constantly growing for several years—in 2016 it was 50.79, while in 2008 it was 44.19. These data confirm that young nurses are the first to leave the profession. Diagnosis of the working conditions and psychosocial burden level among nurses should be subject to detailed analysis, so that leaving the profession will not additionally deepen the difficult staffing situation in health care.

**Aim:**

The aim of the study was to identify factors affecting the assessment of work arduousness levels among nursing personnel.

**Materials and Methods:**

The study was conducted among 573 nurses working on surgical, medical treatment, and emergency wards. A standardized job evaluation questionnaire was used to conduct the survey.

**Results:**

(1) Stress levels depended on the ward in which the surveyed person worked. Nurses working in the emergency ward assessed their conditions the best, with the lowest stress. The average general result in this group was 38.1 points versus 46 and 45.7 points in the surgical and medical treatment wards, respectively. (2) At the level of the whole studied group, both the nurses' age and work experience did not differ statistically significantly in the total assessment of working conditions. Differences in the assessment of work arduousness in different age categories occurred at the level of individual wards. In the surgical ward, younger employees were characterized by higher stress levels, especially in the area of arduousness (*p*=0.0165). In the medical treatment wards, there was a similar age-to-stress ratio for the area of organizational uncertainty (*p*=0.0063). With age, employees of the emergency ward became more indifferent to stress related to unpleasant working conditions (*p*=0.0009), while stress related to organizational uncertainty increased (*p*=0.0495). (3) Nurses working in managerial positions assessed the overall stress related to their job higher than other nurses. They were particularly at risk for burdens related to haste, responsibility, and organizational uncertainty. The average overall assessment of work arduousness for this group was 44.6 points, while for surgical nurses it was 37.2 points. Correlations between the performed function and stress levels were found for almost all of the studied work characteristics (except for hazards). (4) Education had a statistically significant impact on the perception of working conditions in several dimensions. The people with the lowest education evaluated working conditions the best. The difference between people with a higher and those with a secondary education with a specialization was definitely smaller and often nonexistent. Education differentiated the work arduousness assessment depending on the ward. The most statistically significant correlations were obtained in surgical wards, and the least in medical treatment wards.

**Conclusions:**

(1) The study results indicate the need to diagnose problems related to work conditions in the context of occupational stress within individual hospital wards. To limit employee turnover, nursing staff managers should approach the issue of improving working conditions individually for each ward, due to differences in the nature of the work and level of stressogenicity. (2) In each hospital ward, employees at different stages of their career are sensitive to the psychosocial burden resulting from different work characteristics. These areas should be thoroughly diagnosed and the burden minimized to prevent departures from the profession—at early stages of the professional career as well as among experienced personnel. (3) Nurses working in managerial positions should receive the necessary substantive support, due to the higher stress burden associated with greater responsibility.

## 1. Introduction

Staff shortages among nurses have been severely felt in most countries around the world for many years. In Poland, this problem is particularly visible due to the lowest nursing employment rate per 1000 inhabitants among 28 EU states, which in 2015 was merely 5.2. In comparison, in Denmark, it was 16.7, in Germany 13.3, and in France 9.9, while the average for 35 OECD member countries was 9.0 [1].

Research by the Supreme Council of Nurses and Midwives indicates that the shortage of nurses in Poland is much higher than the available studies show. This is due to the fact that the established minimum employment standards are based on the basis of registered health services, taking into account the number of beds, the specificity of the ward, and not the actual needs of the medical facility. The result is one nurse working the night shift or less personnel on Sundays and holidays. This translates into a greater workload due to the increased number of responsibilities per person [2].

Another unfavorable phenomenon observed in this professional group in Poland is the gradual increase in the average age of registered nurses. In 2008, it was 44.19, while in 2016 it was 50.79, which means a rise by 6.6 years in only 8 years. The majority of registered nurses were in the 41–60 age group (66.51%), while nurses in the youngest age group from 21 to 35 constituted only 5.53% of all registered nurses [3].

The successive increase in the average age of nurses and the declining percentage of nurses in the youngest age group may indicate several phenomena, including lack of employment of new nurses in the profession despite obtained qualifications, financially motivated emigration after graduation, as well as leaving the profession early in the career and retraining to work in another profession. The scientific literature has identified a number of factors that are the most frequent reasons for nurses leaving the profession [4–8]. They include factors such as low wages, few career development opportunities, shift work, health problems, and the psychosocial burden in the workplace.

Psychosocial burden, in the case of nurses, concerns, among others, overloading with physical work, fears of infection with diseases, work complexity, the ambiguity of the role played in the organization, unpleasant working conditions, conflicts, employment insecurity, and negative family relations resulting from a work-home conflict. Excessive workload is compounded by the fact that in Poland, as in many other countries in the world, this profession is strongly feminized. As a result, there is no possibility that particularly physically burdensome work, such as lifting patients, for example, can be performed by physically stronger men. In addition, nursing aids, who could perform nursing activities around the patient, are not employed in Poland. Therefore, in addition to providing medical care, nurses perform a number of activities, such as patient hygiene and washing, change of bed linens, transporting patients for tests, and other activities [9]. The high physical burden and resulting fatigue additionally intensify stress, the fear of making a mistake, which in the case of nurses can have far-reaching consequences (giving the patient the wrong dose of a drug, for example). Furthermore, the nature of the work causes a rise in the number of factors that are hazardous to nurses' health, including those that require lifting, walking with a heavy load, and taking a forced body position. [10, 11].

The complexity of work in the case of nurses is first and foremost the necessity to perform many activities requiring attention and accuracy, such as administering the right doses of medicines, carrying out measurements (e.g., blood pressure), handling medical devices that are interlaced with hard physical work. A significant source of stress is conflict at the workplace, which include conflicts with other nurses and superiors, including frequent cases of bullying [12, 13].

The health problems of nurses often originate in psychosocial burdens at the workplace. This is confirmed by numerous scientific studies on the general impact of stress on the physical and mental health of employees as well as in detailed research, e.g., confirming the unequivocally negative impact of bullying on psychoemotional aspects of a nurse's health, and indirectly as one of the factors causing burnout on the general health condition of nurses [14].

In this study, we attempted to identify the most arduous and most frequently occurring burdens at the workplace of nursing personnel. Using statistical analysis, we diagnosed the effect of particular factors, such as age, duration of professional experience, and position held, on the intensity of psychosocial burden perception and compared the differences in the work arduousness assessment depending on the respondent's place of work, that is, the ward where he/she worked.

## 2. Materials and Methods

The study was conducted from September 2017 to December 2017 in Poland in the Podlaskie Voivodeship. It included 573 people working as nurses at inpatient health care facilities. Participation in the study was voluntary and anonymous. Participants could quit the survey at any level. All procedures were prepared according to the ethics standard approved by the Local Bioethics Committee of the Medical University of Bialystok (ref. no R-I 002/296/2017).

### 2.1. Study Procedure

The study was conducted by a group of experts composed of representatives of nurses and teachers in the nursing profession. These people understood the purpose of the study and knew the specificity of working as a unit nurse at inpatient health care facilities. The study was conducted using a standardized Work Features Assessment Questionnaire developed by Dudek et al. [15]. The questionnaire was developed by a team of Polish researchers and therefore it was considered the best suited tool to the specifics of the studied population. The experts thoroughly explained the purpose and meaning of the individual questions to the study participants and then filled out the questionnaire themselves based on the respondents' responses and observations. This way of conducting the study provided an objective assessment of work stressfulness. “Objectivity” in this case means that the assessment was not dependent on the individual stress experienced by the respondent and is a resultant of assessments made independently by 2-3 experts familiar with the specificity and working conditions at a given position.

### 2.2. Study Group Selection

The selection of respondents to the research group was based on the register of nurses associated with the District Chamber of Nurses and Midwives in Białystok. The criterion was employment based on a contract of employment at a hospital in medical treatment, surgical, or emergency units. 10% of randomly selected persons meeting the selection criterion were invited to the study.

### 2.3. Description of the Questionnaire and the Applied Measures

A standardized Work Features Assessment Questionnaire for objective assessment of work stressfulness was used as a research tool [15]. The questionnaire consisted of 34 statements describing particular work features. These statements were rated on a scale of 1 to 5 depending on the feature's frequency, duration, or severity.

Based on the statements of the questionnaire, 10 specific measures were determined: unpleasant working conditions, work complexity, hazards, conflicts, uncertainty resulting from the organization of work, arduousness, haste, responsibility, physical effort, competition, and one overall measure of work arduousness. The higher the score, the higher the work arduousness in a given aspect. The results for the individual measures are not comparable with each other, because each individual measure (and overall measure) has a different number of component statements. To allow for comparison between work arduousness estimations in different categories, raw values of the individual measures were normalized to a range of 0–100, with 0 indicating the absence of work arduousness and 100 indicating the maximum work arduousness.

Based on the standards set out in the questionnaire, individuals with high stress levels due to work arduousness in the different areas were distinguished. In the case of the overall scale of work arduousness, three categories were distinguished: low, medium, and high.

### 2.4. Statistical Methods

Statistical analysis was performed using the appropriate statistical tests, by means of which the statistical significance of the considered dependencies was verified. In the case of studying the effect of a nominal factor (such as the ward) on work arduousness numerical values, descriptive statistics were determined in the compared groups and differences in the distribution of the measures between the groups were assessed using the Mann–Whitney test (for two groups) or the Kruskal–Wallis test (for three or more compared groups). When studying the effect of a numerical feature (e.g., age or work experience) on work arduousness numerical values, Spearman's rank correlation analysis was used. The percentage of people with a high level of work arduousness in particular professional areas relative to the grouping factor was compared using the chi-square test.

## 3. Results

The study included 573 respondents working as nurses. The vast majority of the respondents were women (97%). The average age for the studied group was 38.5 years, with a slightly higher median equal to 39 years. The youngest employee was 21 years old and the oldest was 61. Over half of the respondents had completed nursing studies, one-fourth had a secondary education with a specialization, and one-fifth had secondary education. The average duration of work experience was 15 years, with a slightly lower median of 14 years. Work experience ranged from one to 41 years. The majority (3/4 of the respondents) worked as a unit nurse. Every tenth nurse was employed as a surgical nurse, and every twentieth held a managerial position. The percentage distribution of employees between wards (surgical, medical treatment, emergency) was almost even.

**Figure 1:**
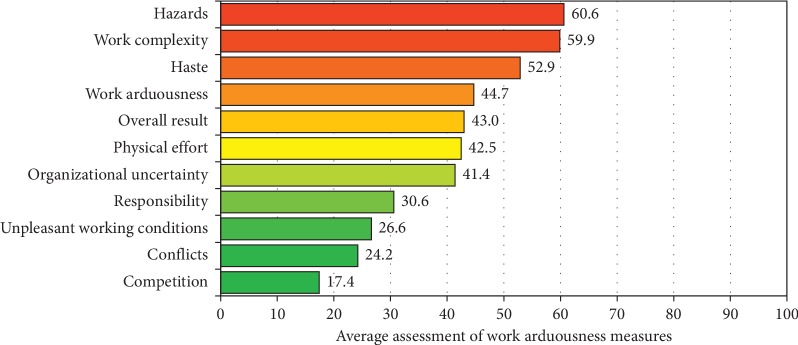
Ranking of work arduousness measures.

On the basis of the obtained results, a ranking of work arduousness measures, presented in Figure 1, was prepared. The elements of work such as hazards, work complexity, and haste were defined as the most arduous, and the least problems occurred in such categories as conflicts or competition.

**Figure 2:**
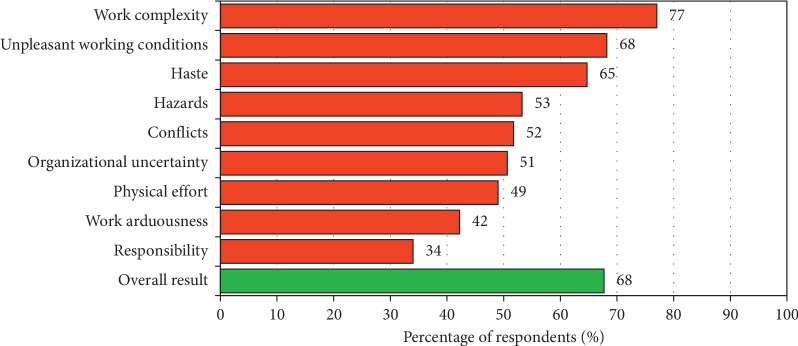
Ranking of work arduousness in terms of frequency of occurrence.

Next, based on the standards set out in the questionnaire [12], individuals with high stress levels due to work arduousness in the different areas were distinguished, which allowed determining the burden intensity of particular elements of work. The ranking of the elements of work in terms of frequency of occurrence to a degree causing high stress levels is shown in Figure 2. Most often, high levels of stress were caused by work complexity, unpleasant working conditions, and haste, and the least often by arduousness and responsibility.

In the case of the overall scale of work arduousness, three categories were distinguished: low, medium, and high. According to such a classification, more than two-thirds of the respondents considered their work to be very arduous, and only one in twenty to be easy.

The work arduousness values were compared in terms of the ward on which the respondents worked. The results of the comparison lead to an unambiguous conclusion about the significant impact of this factor on work assessment. For all areas in which work arduousness was assessed, the differences between wards were statistically significant. Considering the total work arduousness levels, the emergency ward stood out (the average level for the general result in this group was 38.1 points versus 46 and 45.7 points for surgical and medical treatment wards, respectively). Detailed data are presented in Table 1, which shows that the emergency ward was characterized by the lowest stress levels in all areas. In all wards, such features as hazards, haste, and work complexity were rated as the most arduous, whereas competition, conflicts, and unpleasant working conditions as the least arduous.

**Figure 3:**
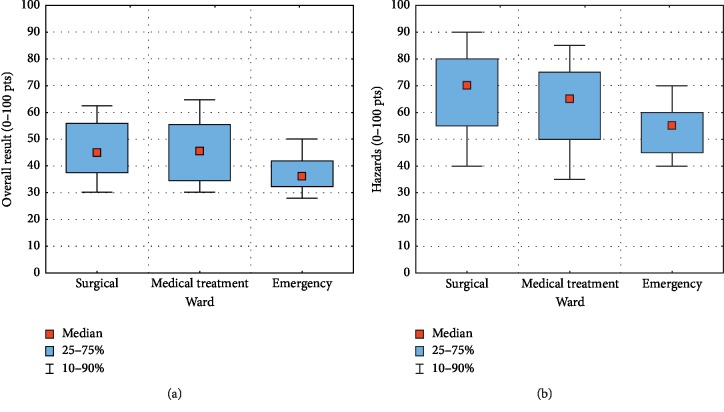
Median, quartiles, and centiles for overall results and hazards.

Figure 3, a box and whisker plot, shows the median level, the values of the 25th and 75^th^, as well as the 10th and 90th centile of work arduousness measures for the overall result and the hazards measure (selected as an example from the other measures).

The relationship between the ward and work arduousness occurrence was also compared in terms of the incidence of people who felt high burden levels in individual areas. In this analytical approach, we also found highly statistically significant differences between the wards for almost all the considered areas (with the exception of the work complexity category). Analyzing the results in Table 2 in detail, it can be stated that the smallest percentage of people experiencing high work arduousness occurred in the emergency ward and the largest in the surgical ward. The most intense on all wards were features such as haste, unpleasant working conditions, conflicts, and organizational uncertainty. Additionally, organizational uncertainty occurred most frequently in the case of medical treatment wards and hazards in the case of surgical wards. The least intense were responsibility and work arduousness and physical effort, only in the case of surgical wards.

Analysis of the variation of work arduousness assessments in terms of the wards ends with a list of adjective distribution of the arduousness scale in the compared groups, listed in Table 3. Differences in the assessment of stressful situation occurrence between respondents from individual wards were quite significant. For example, on surgical wards, as many as 81% of the employees assessed work as highly stressful, whereas on the emergency wards just over 50% gave such an answer. Differences in the distribution of the classification of stress levels at work between the considered wards are statistically significant.

The effect of age on the assessment of stress levels at work in particular areas and on the overall result was examined. The analysis consisted of determining the Spearman's rank correlation coefficients between age and the numerical measures of stressful situation occurrence, determined on the basis of a standardized questionnaire. The analysis was carried out both at the level of the entire population and when controlling for the ward type, because this factor may affect the occurrence of dependence (as previously noted, stress levels depended on the type of ward in which the respondents worked).

At the level of the entire surveyed population, very weak correlations with age were found only in two areas: haste and physical effort. The stressfulness of haste accompanying work increased with age and the stress associated with physical effort decreased with age. However, both of these correlations had negligible strength (Table 4), which means that their practical meaning is almost none.

An alternative form of analysis was also conducted. We divided the respondents into four age groups, presenting the descriptive statistics values in these groups and assessing the differences between them using the Kruskal–Wallis test. This analysis ignored age differences within the created groups, which may lead to different results than the conducted correlation analysis. As shown in Table 5, the created groups were quite numerous, which allowed for reliable analyses.

Analysis of work stressfulness in relation to age groups led to distinguishing one highly significant result; namely, age differentiates the assessment of stress resulting from physical effort. The stress levels associated with this were much higher among employees aged up to 39 years (median 50 points) compared with other people (median 37.5 points). Other burdens were not correlated with age in any way (Table 6).

A similar correlation analysis was performed for each ward. It turned out that this was the right approach, because we found that there were more statistically significant relationships within individual wards. These were also dependencies of slightly greater strength than the two correlations found at the level of the entire population. The analysis results are presented in Table 7. Younger employees in the surgical wards were characterized by higher stress levels. This pertains to areas such as arduousness, responsibility, competition, and the overall result. On the medical treatment wards, there was a similar (to the surgical ward) age-to-stress ratio for the areas of organizational uncertainty and physical effort. With age, employees of emergency wards became more indifferent to stress related to unpleasant working conditions, hazards, and physical effort, while stress related to organizational uncertainty increased.

Work experience was very strongly correlated with age (Spearman's rank correlation coefficient between these two features was *R* = 0.94), so it could be expected that the duration of work experience would affect the work stress assessment in a similar way as age. The results in Table 8 confirm this assumption. Only two statistically significant, but very weak, correlations can be found at the level of the entire population—between work experience and the stressfulness of haste (those with more work experience were more susceptible to this factor) and the level of stress induced by physical effort (here, for a change, more work experience was a positive factor in the “immunity” of an employee to physical effort). Both of these correlations had very little strength.

Taking into account the specifics of individual wards in the analysis leads to much more interesting results. Work experience was a factor that had the most significant impact on the stress levels of surgical ward employees. All the distinguished, statistically significant correlations had a negative sign, which means that people with more work experience were more resistant to the occurrence of stressful situations at work. Work experience affected the stress caused by work burdens, responsibility, and the overall result the strongest. Among employees of surgical wards, more work experience had a positive effect (stress levels decreased) on only two areas: organizational uncertainty and physical effort, whereas, among emergency ward employees, longer work experience increased stress levels caused by conflicts, organizational uncertainty, work arduousness, and haste and decreased the stress associated with unpleasant working conditions and physical effort (Table 9).

The relationship between the held position and the assessment of work arduousness was analyzed.  Table 10 presents the values of basic descriptive statistics and the significance assessment of differences in the stress levels of employees depending on the performed function. Starting the interpretation from the general stress level, we noted that it was higher among nurses who performed managerial functions and among unit nurses. Nurses in management were particularly vulnerable to stress associated with haste, work complexity, organizational uncertainty, or work arduousness. Very weak correlations were found in the case of unpleasant working conditions, conflicts, and competition. The only area in which we found no effect of the held position on stress levels was the occurrence of hazards.

Due to the fact that ward type was a factor strongly differentiating assessment of working conditions, analysis of the impact of education on working conditions was done separately for each ward type. To assess the significance of the differences between the groups the Kruskal–Wallis test was used.

In the group of nurses working in surgical wards, education had a statistically significant impact on the perception of working conditions in several dimensions. The people with the lowest education evaluated working conditions the best. The difference between people with a higher and those with a secondary education with a specialization was definitely smaller, and often assessments of working conditions in these two groups were almost identical (Table 11).

Among people working on medical treatment wards, the impact of education on the assessment of working conditions was not as pronounced. Only the assessment of stressogenicity related to responsibility and physical effort differed in a statistically significant way due to the education level. In this first dimension (responsibility), people with higher education had the highest stress levels. In the second dimension (physical effort), the differences were not so clear and logically oriented, so it is difficult to interpret these results unambiguously.

From the nurses working on the emergency ward, education differentiated the assessment of unpleasant working conditions, work complexity, and uncertainty resulting from the organization of work. In the first two areas, the higher a nurse's education, the worse the assessment of that particular dimension. In terms of organizational uncertainty, the worst assessments were obtained from nurses with a secondary education with a specialization.

Finally, a multivariate analysis was done. A regression model was constructed for the overall assessment of work conditions, in which, apart from nominal factors, which were ward and education, the potential impact of age and work experience was also considered. These variables had numerical values, so they were treated as continuous variables. The models also considered the 2nd-degree interactions between all factors. Using the stepwise regression procedure, the optimal model was selected. This model included only nominal factors: ward (*p*=0.0000^*∗∗∗*^) and education (*p*=0.0001^*∗∗∗*^); the interaction between them was also significant (*p*=0.0185^*∗*^). The nurses' work experience and age did not differentiate in a statistically significant way the total assessment of working conditions.

**Figure 4:**
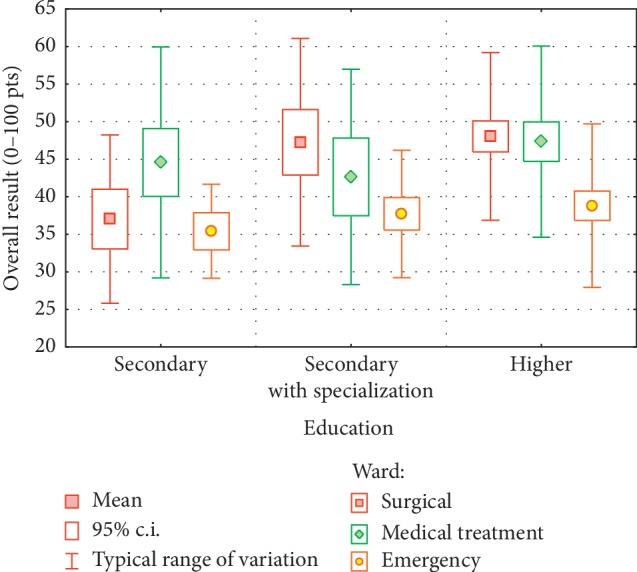
Overall assessment of work conditions.

Since only two nominal factors remained in the model, the results can be described in terms of analysis of variance, presenting the values of descriptive statistics in the compared groups.  Table 12 presents average values and standard deviation. To facilitate the interpretation of the results, a graphic presentation (Figure 4) of group averages with a 95% confidence interval and a typical range of variation was also included. Analyzing the distribution of group averages, we can state that

- People with a higher education assessed working conditions more negatively
- Nurses working in the emergency ward assessed their conditions the best and assessed their stress levels the lowest
- The effect of education on the working conditions assessment depended on the ward, with the largest differences in assessments occurring on the surgical ward

## 4. Discussion

Numerous studies conducted in different countries have shown that working as a nurse is characterized by clearly higher stress levels than the average stress levels for the employed population [16]. Poland has one of the lowest nursing employment rates (5.2 per 1000 residents) in Europe and a high rate of leaving the profession. The average age of Polish nurses has been constantly growing for several years—in 2016 it was 50.79, while in 2008 it was 44.19. These data confirm that young nurses are the first to leave the profession. It is similar in other European countries, such as Italy [17], Finland [18], and Sweden [19]. Many studies from different countries have shown that in addition to such factors as low wages or labor migration, working conditions have a huge impact on the number of nurses leaving the profession [20–25].

In this article, we decided to examine which factors affect the assessment of work arduousness among the nursing staff and whether there are determinable correlations between them. There have been many studies on stress [26] and recommendations on what measures should be taken to minimize the negative effects of stress. The majority of studies treated nurses as a homogeneous group, regardless of age, work experience, place of work, and sex, or these studies were conducted within a specific ward, such as intensive care or emergency. For our study, we selected three types of hospital departments—surgical, medical treatment, and emergency wards—to compare whether the results within individual wards differ from those obtained for the entire studied population. This was to verify whether individual work conditions, characteristics of individual wards, affect the assessment of work stressfulness.

Study results showed that at the level of the whole surveyed group, the nurses' age, education, and work experience did not differ statistically significantly in the total assessment of working conditions. Differences in the assessment of work arduousness levels in different age categories occurred at the level of individual wards. Similar results were also obtained in the case of work experience, education, and the position held. All these factors correlated differently depending on the ward, and even in many cases, any correlation occurred within the ward and did not occur in the entire studied population.

Similarly, research conducted in Iran showed a lack of correlations at the level of the whole group between demographic factors (such as age, sex, education, work experience) and the level of work satisfaction, which was strongly related to stress levels [27]. Studies conducted in Sudan in public hospitals in Khartoum State [28] and in private and public hospitals in Amman, Jordan [29] showed that the psychosocial burden felt by nurses varied depending on the ward, similarly to our results. Research on work satisfaction among nurses in Great Britain also indicated the need to conduct analyses at the level of individual hospital departments [25]. Our research showed not only differences in the psychosocial burden depending on the ward, but also other correlations between age, work experience, and education and psychosocial factors occurring within various ward types.

Keeping in mind the earlier observation that the youngest nurses most often leave the profession, we examined how age and experience affected the nurses' perception of psychosocial burdens. The obtained results indicate that with age, employees become more immune to certain stressors. On the surgical wards, the most noticeable were arduousness, responsibility, and competition for young employees; on medical treatment wards, uncertainty resulting from the organization of work and physical effort; while on the emergency ward, unpleasant working conditions, hazards, and physical effort. A similar effect was observed in the case of work experience. Employees with the least work experience felt burdens almost identically to the youngest employees, except for the emergency ward, where no correlations were found in the case of hazards, while inverse correlations were found in the case of uncertainty resulting from the organization of work and haste. These two factors in the case of employees of emergency wards increased with experience.

This can be explained by the fact that with age, and thus with increased experience, some factors become less and less stressful, probably due to the fact that employees somehow get used to certain working conditions and do not perceive them as negatively as initially. Research conducted at the Medical University of Gdańsk among nurses employed in hospitals [30], outpatient clinics, and social care homes showed, similarly to our results, the highest levels of psychosocial burden among the youngest nurses. Studies carried out in Italian hospitals [31] showed that good workplace conditions positively stimulated a decline in work efficiency progressing with age, whereas a study conducted in four hospitals in Poland showed that nurses over 40 had the highest emotional exhaustion rate [32]. This is quite different from our study results.

An important result of the conducted research is also an indication of the burdens that did not depend on the nurses' age or experience. Regardless of the ward where the nurses worked, work complexity, conflicts, and haste were felt regardless of age, and hazards regardless of experience.

Organizational uncertainty, or a feeling of a threat of job loss, is a more stressful factor than the loss of work alone [33]. This factor intensified with age and work experience. This was confirmed by our research in the case of the emergency ward, and a completely different result was obtained for the medical treatment ward, where this factor decreased with age.

The psychosocial burden that nurses experience in their daily work may be the cause of their mistakes (incorrect doses, inappropriate medicines). This fact was confirmed by research conducted in public hospitals in Tehran, which showed a close correlation between the stress experienced by nurses and the number of mistakes made during treatment [34]. Similar results were obtained in India [35], as well as in Canada [36], showing a correlation between stress levels and the number of mistakes, injuries, and negligence.

The psychosocial burden had an impact on general job satisfaction among nurses, which in turn leads to employees being more likely to start looking for other career opportunities [37]. This is particularly important in the context of our obtained results, according to which the assessment of work arduousness of nurses working in managerial positions and those better educated was worse than other nurses. Nurses employed in managerial positions in the entire studied population assessed their workplace worse in all the assessed areas, with the exception of hazards. Education differentiated the assessment depending on the ward. We identified the presence of the most features on surgical wards: work complexity, arduousness, haste, responsibility, and physical effort whereas on medical treatment wards only responsibility and physical effort and on emergency wards unpleasant working conditions and work complexity. On the basis of such results, we can draw two conclusions. First, nurses with higher education and employed in managerial positions assess their working conditions worse, because they are usually burdened with more responsibility and a broader scope of duties. Second, thanks to their education, and usually more work experience, they are more aware of the hazards in the workplace.

One of the limitations of the conducted research is that we show a static picture of the psychosocial burdens in the nurses' workplace. An interesting issue seems to be the impact of global economic changes on the dynamics of psychosocial burdens in the workplace. The negative impact of the global crisis on workers' health was confirmed in the results of research carried out in Northern Ireland [38].

Summing up the results of our research, we found that the factors that influence the assessment of the nurses' working conditions were the position and type of ward they were employed in. Age, work experience, and education did not have a statistically significant impact on the assessment of nurses' working conditions if we treated the nurses as a homogeneous group. The results changed radically if we conducted analyses within the ward type. We then found statistically significant dependencies of the assessment of working conditions depending on age, work experience, education, and position held. In all the wards, the youngest employees were the most exposed to stress, but the most stressful was other work features in each ward. In the surgical ward, these were arduousness, responsibility, and competition; in the medical treatment ward: organizational uncertainty and physical effort; and in the emergency ward: unpleasant working conditions, hazards, and physical effort. Little work experience intensifies stress in the surgical wards, especially in terms of arduousness and responsibility; in the medical treatment ward: organizational uncertainty and physical effort; and unpleasant working conditions and physical effort in the emergency ward. Only in the emergency ward, as many as four features—organizational uncertainty, haste, arduousness, and conflicts—were perceived worse by employees with more work experience. Higher education affected a more critical assessment of the working conditions, which, however, differed between the wards. In the surgical ward, people with higher education experienced work complexity, arduousness, and haste as the worst; in the medical treatment ward, it was responsibility and physical effort; and in the emergency ward, unpleasant working conditions and work complexity.

In the face of staff shortages among nurses, which are intensifying due to the aging of society, it is necessary to diagnose factors that increase the stressfulness of work, so that effective actions to counteract them can be taken. Particular attention should be paid to young people, with less work experience and better education, as they are the most susceptible to the psychosocial burden and leave the profession the most often.

## 5. Conclusions

1. The study results indicate the need to diagnose problems related to work conditions in the context of occupational stress within individual hospital wards. To limit employee turnover, nursing staff managers should approach the issue of improving working conditions individually for each ward, due to differences in the nature of the work and level of stressogenicity.
2. In each hospital ward, employees at different stages of their career are sensitive to the psychosocial burden resulting from different work characteristics. These areas should be thoroughly diagnosed and the burden minimized to prevent departures from the profession—at early stages of the professional career as well as among experienced personnel.
3. Nurses working in managerial positions should receive the necessary substantive support, due to the higher stress burden associated with greater responsibility.

## Figures and Tables

**Table 1:** Work arduousness values on the wards.

Work features assessment (0–100 pts)	Ward									*p*
	Surgical			Medical treatment			Emergency			
	$\bar{x}$	Me	*s*	$\bar{x}$	Me	*s*	$\bar{x}$	Me	*s*	
Unpleasant working conditions	31.7	30.0	17.0	23.4	15.0	21.6	24.5	25.0	12.7	≤0.001∗∗∗
Work complexity	60.3	62.5	16.2	63.8	66.7	16.9	56.3	58.3	14.4	≤0.001∗∗∗
Hazards	67.3	70.0	17.5	61.3	65.0	19.3	53.9	55.0	14.1	≤0.001∗∗∗
Conflicts	22.5	18.8	16.3	30.2	25.0	19.5	20.8	18.8	13.8	≤0.001∗∗∗
Organizational uncertainty	45.1	37.5	26.2	47.7	50.0	28.0	33.0	31.3	21.2	≤0.001∗∗∗
Work arduousness	51.8	50.0	18.5	44.7	41.7	20.3	38.3	41.7	16.7	≤0.001∗∗∗
Haste	57.2	62.5	25.5	56.5	62.5	23.5	46.0	50.0	21.1	≤0.001∗∗∗
Responsibility	31.9	25.0	33.3	37.0	37.5	28.6	24.4	12.5	23.4	0.0002∗∗∗
Physical effort	42.6	50.0	19.2	44.9	50.0	18.4	40.5	37.5	15.3	0.0217∗
Competition	16.0	0.0	28.3	25.1	0.0	34.0	12.3	0.0	21.7	0.0025∗∗
Overall result	46.0	44.9	12.4	45.7	45.6	13.8	38.1	36.0	9.8	≤0.001∗∗∗

*p* value of statistical significance calculated using the Kruskal–Wallis test.

**Table 2:** Work arduousness frequency of occurrence in the wards.

Work features assessment (0–100 pts)	Ward						*p*
	Surgical		Medical treatment		Emergency		
	*N*	%	*N*	%	*N*	%	
Unpleasant working conditions	150	79.4	82	47.7	159	75.0	≤0.001^*∗∗∗*^
Work complexity	143	75.7	141	82.0	157	74.1	0.1629
Hazards	136	72.0	100	58.1	69	32.5	≤0.001^*∗∗∗*^
Conflicts	87	46.0	108	62.8	101	47.6	0.0021^*∗∗*^
Organizational uncertainty	107	56.6	108	62.8	75	35.4	≤0.001^*∗∗∗*^
Work arduousness	118	62.4	63	36.6	61	28.8	≤0.001^*∗∗∗*^
Haste	140	74.1	122	70.9	109	51.4	≤0.001^*∗∗∗*^
Responsibility	69	36.5	76	44.2	50	23.6	≤0.001^*∗∗∗*^
Physical effort	97	51.3	99	57.6	85	40.1	0.0023^*∗∗*^
Overall result	153	81.0	126	73.3	109	51.4	≤0.001^*∗∗∗*^

*p* value of statistical significance calculated using the chi-square test.

**Table 3:** Distribution of the arduousness scale in groups.

Overall result	Ward (*p* ≤ 0.001^*∗∗∗*^)			Total
	Surgical	Medical treatment	Emergency	
Low	9 (4.8%)	13 (7.6%)	7 (3.3%)	29
Medium	27 (14.3%)	33 (19.2%)	96 (45.3%)	156
High	153 (81.0%)	126 (73.3%)	109 (51.4%)	388
Total	189	172	212	573

*p* value of statistical significance calculated using the chi-square test.

**Table 4:** Impact of age on the assessment of the working conditions.

Work features assessment (0–100 pts)	Age (years)
Unpleasant working conditions	−**0.08** (*p*=0.0501)
Work complexity	−0.03 (*p*=0.4040)
Hazards	−0.03 (*p*=0.5228)
Conflicts	0.03 (*p*=0.4827)
Organizational uncertainty	0.01 (*p*=0.8987)
Work arduousness	−0.01 (*p*=0.8884)
Haste	**0.09 (** *p*=0.0356^*∗*^**)**
Responsibility	−0.03 (*p*=0.4945)
Physical effort	−**0.11** (*p*=0.0063^*∗∗*^)
Competition	−**0.08** (*p*=0.0670)
Overall result	−0.03 (*p*=0.4553)

**Table 5:** Age groups.

Age (years)	Number	Percent
<30	141	24.7
30–39	158	27.7
40–49	183	32.0
>50	89	15.6

**Table 6:** Assessment of working conditions within age groups.

Work features assessment (0–100 pts)	Age (years)												*p*
	<30			30–39			40–49			>50			
	$\bar{x}$	Me	*s*	$\bar{x}$	Me	*s*	$\bar{x}$	Me	*s*	$\bar{x}$	Me	*S*	
Unpleasant working conditions	28.5	25.0	17.4	26.9	25.0	18.2	25.7	25.0	16.5	25.1	20.0	18.6	0.2925
Work complexity	60.4	58.3	16.2	61.1	62.5	15.2	58.0	58.3	16.4	60.4	62.5	16.5	0.2249
Hazards	61.1	60.0	18.9	61.4	60.0	17.2	59.9	60.0	17.1	59.5	55.0	18.8	0.8362
Conflicts	24.3	25.0	16.9	22.9	18.8	17.0	25.2	25.0	17.4	23.9	25.0	16.5	0.6759
Organizational uncertainty	41.4	37.5	28.0	41.7	31.3	26.2	41.9	37.5	24.3	40.0	31.3	25.6	0.9346
Work arduousness	44.7	41.7	21.0	44.4	41.7	18.4	44.9	41.7	19.1	45.1	41.7	18.5	0.9935
Haste	49.2	50.0	23.6	53.7	50.0	24.0	54.8	50.0	25.1	53.5	62.5	21.4	0.1183
Responsibility	28.3	25.0	26.7	36.2	37.5	31.0	29.6	25.0	28.4	26.7	12.5	28.8	0.1244
Physical effort	**44.9**	**50.0**	**15.2**	**44.5**	**50.0**	**19.0**	**39.5**	**37.5**	**18.0**	**41.6**	**37.5**	**17.7**	**0.0096∗∗**
Competition	19.3	0.0	28.6	18.4	0.0	29.1	16.5	0.0	28.3	14.6	0.0	28.2	0.2429
Overall result	43.3	41.9	12.9	43.7	41.9	12.0	42.5	39.0	12.4	42.2	39.7	13.4	0.7239

*p* value of statistical significance calculated using the Kruskal–Wallis test.

**Table 7:** Impact of age on the assessment of the working conditions by ward.

Work features assessment (0–100 pts)	Ward		
	Surgical	Medical treatment	Emergency
	Age (years)		
Unpleasant working conditions	0.00 (*p*=0.9853)	−0.01 (*p*=0.8684)	−**0.23** (*p*=0.0009^*∗∗∗*^)
Work complexity	−0.09 (*p*=0.2029)	−0.03 (*p*=0.6933)	−0.11 (*p*=0.1190)
Hazards	−0.08 (*p*=0.2608)	−0.05 (*p*=0.4879)	−**0.16** (*p*=0.0222^*∗*^)
Conflicts	−0.12 (*p*=0.1099)	−0.01 (*p*=0.8970)	0.10 (*p*=0.1600)
Organizational uncertainty	−0.04 (*p*=0.5686)	−**0.21** (*p*=0.0063^*∗∗*^)	**0.14** (*p*=0.0495^*∗*^)
Work arduousness	−**0.17** (*p*=0.0165^*∗*^)	−0.01 (*p*=0.8649)	0.09 (*p*=0.1981)
Haste	0.06 (*p*=0.4196)	−0.01 (*p*=0.9262)	0.11 (*p*=0.1089)
Responsibility	−**0.15** (*p*=0.0365^*∗*^)	−0.08 (*p*=0.3181)	0.06 (*p*=0.3982)
Physical effort	−0.10 (*p*=0.1880)	−**0.17** (*p*=0.0242^*∗*^)	−**0.15** (*p*=0.0285^*∗*^)
Competition	−**0.13** (*p*=0.0660)	−0.13 (*p*=0.1002)	−0.03 (*p*=0.6918)
Overall result	−**0.13** (*p*=0.0729)	−0.11 (*p*=0.1528)	−0.03 (*p*=0.7138)

**Table 8:** Impact of work experience on the assessment of the working conditions.

Work features assessment (0–100 pts)	Work experience (years)
Unpleasant working conditions	−0.04 (*p*=0.3033)
Work complexity	−0.04 (*p*=0.3854)
Hazards	0.00 (*p*=0.9654)
Conflicts	0.02 (*p*=0.6646)
Organizational uncertainty	0.03 (*p*=0.5093)
Work arduousness	0.01 (*p*=0.8019)
Haste	**0.10** (*p*=0.0157^*∗*^)
Responsibility	−0.04 (*p*=0.2995)
Physical effort	−**0.13** (*p*=0.0028^*∗∗*^)
Competition	−**0.07** (*p*=0.0825)
Overall result	−0.01 (*p*=0.7379)

**Table 9:** Impact of work experience on the assessment of the working conditions by wards.

Work features assessment (0–100 pts)	Ward		
	Surgical	Medical treatment	Emergency
	Work experience (years)		
Unpleasant working conditions	−0.04 (*p*=0.6275)	0.00 (*p*=0.9823)	−**0.16 (***p*=0.0199^*∗*^**)**
Work complexity	−**0.15 (***p*=0.0363^*∗*^**)**	−0.03 (*p*=0.7224)	−0.07 (*p*=0.3274)
Hazards	−0.10 (*p*=0.1925)	−0.05 (*p*=0.5337)	−0.11 (*p*=0.1119)
Conflicts	−**0.15 (***p*=0.0413^*∗*^**)**	−0.02 (*p*=0.8190)	**0.13 (** *p*=0.0545**)**
Organizational uncertainty	−0.06 (*p*=0.4512)	−**0.18 (***p*=0.0244^*∗*^**)**	**0.17 (** *p*=0.0108^*∗*^**)**
Work arduousness	−**0.26 (***p*=0.0003^*∗∗∗*^**)**	0.04 (*p*=0.6043)	**0.14 (** *p*=0.0453^*∗*^**)**
Haste	−0.01 (*p*=0.8879)	0.04 (*p*=0.6074)	**0.16 (** *p*=0.0176^*∗*^**)**
Responsibility	−**0.19 (***p*=0.0111^*∗*^**)**	−0.06 (*p*=0.4128)	0.07 (*p*=0.3459)
Physical effort	−**0.12 (***p*=0.0975**)**	−**0.18 (***p*=0.0213^*∗*^**)**	−**0.15 (***p*=0.0274^*∗*^**)**
Competition	−**0.16 (***p*=0.0275^*∗*^**)**	−0.12 (*p*=0.1409)	0.02 (*p*=0.7978)
Overall result	−**0.20 (***p*= 0.0076^*∗∗*^**)**	−0.09 (*p*=0.2739)	0.04 (*p*=0.5309)

**Table 10:** Impact of performed function on the assessment of the working conditions.

Work features assessment (0–100 pts)	Function												*p*
	Managerial			Ward nurse			Surgical nurse			Other			
	$\bar{x}$	Me	*s*	$\bar{x}$	Me	*s*	$\bar{x}$	Me	*s*	$\bar{x}$	Me	*s*	
Unpleasant working conditions	19.1	15.0	20.1	27.0	25.0	18.0	26.4	25.0	14.8	27.0	30.0	12.3	0.0221∗
Work complexity	64.1	64.6	16.0	61.2	62.5	15.4	47.7	43.8	16.3	59.1	62.5	16.0	≤0.001∗∗∗
Hazards	57.7	55.0	22.3	61.2	60.0	17.7	59.8	65.0	17.9	55.8	55.0	13.9	0.3293
Conflicts	29.7	31.3	14.8	24.6	25.0	17.1	20.5	12.5	18.2	20.7	25.0	13.2	0.0114∗
Organizational uncertainty	48.9	43.8	24.7	42.7	37.5	26.1	36.9	31.3	23.7	27.6	18.8	22.4	≤0.001∗∗∗
Work arduousness	46.1	41.7	14.8	46.1	41.7	19.9	34.3	33.3	16.8	42.5	41.7	11.4	≤0.001∗∗∗
Haste	59.8	62.5	24.6	54.6	50.0	23.2	43.5	50.0	25.9	41.4	37.5	22.0	≤0.001∗∗∗
Responsibility	39.3	25.0	34.8	32.4	37.5	28.7	24.8	12.5	27.3	12.2	0.0	20.2	≤0.001∗∗∗
Physical effort	40.6	37.5	15.1	44.3	50.0	17.0	32.1	25.0	22.5	38.5	37.5	12.8	≤0.001∗∗∗
Competition	16.1	0.0	22.8	18.5	0.0	28.9	13.4	0.0	27.8	11.2	0.0	27.7	0.0456∗
Overall result	44.6	41.9	11.5	44.0	41.9	12.5	37.2	33.1	12.4	37.8	37.5	11.1	≤0.001∗∗∗

*p*—value of statistical significance calculated using the Kruskal–Wallis test.

**Table 11:** Impact of education on the assessment of the working conditions of nurses on surgical wards.

Work features assessment (0–100 pts)	Education									*p*
	Secondary (*N* = 33)			Secondary with specialization (*N* = 41)			Higher (*N* = 115)			
	$\bar{x}$	Me	*s*	$\bar{x}$	Me	*s*	$\bar{x}$	Me	*s*	
Unpleasant working conditions	30.0	30.0	17.1	30.5	30.0	16.0	32.7	35.0	17.5	0.1652
Work complexity	**49.9**	**50.0**	**14.1**	**62.8**	**66.7**	**14.9**	**62.4**	**62.5**	**16.1**	**≤0.001** ^*∗∗∗*^
Hazards	62.4	65.0	15.3	68.8	65.0	19.1	68.2	70.0	17.4	0.1536
Conflicts	16.5	12.5	13.2	24.2	18.8	15.7	23.6	18.8	17.0	0.0623
Organizational uncertainty	34.7	37.5	19.6	46.6	43.8	24.8	47.5	37.5	27.8	0.0793
Work arduousness	**39.1**	**33.3**	**15.2**	**50.6**	**58.3**	**21.3**	**55.9**	**58.3**	**16.6**	**≤0.001** ^*∗∗∗*^
Haste	**35.2**	**25.0**	**24.9**	**58.2**	**62.5**	**22.1**	**63.2**	**75.0**	**23.4**	**≤0.001** ^*∗∗∗*^
Responsibility	15.5	0.0	28.1	35.4	37.5	34.4	35.3	25.0	33.1	0.0035^*∗∗*^
Physical effort	**32.2**	**25.0**	**18.0**	**47.0**	**50.0**	**20.1**	**44.0**	**50.0**	**18.3**	**≤0.001** ^*∗∗∗*^
Competition	9.8	0	25.0	17.1	0	28.2	17.4	0	29.3	0.1395
Overall result	**37.0**	**33.1**	**11.2**	**47.3**	**49.3**	**13.8**	**48.0**	**47.1**	**11.2**	**≤0.001** ^*∗∗∗*^

*p*—value of statistical significance calculated using the Kruskal–Wallis test.

**Table 12:** Overall assessment of work conditions.

Education	Overall assessment of work conditions					
	Ward					
	Surgical		Medical treatment		Emergency	
	$\bar{x}$	*s*	$\bar{x}$	*s*	$\bar{x}$	*s*
Secondary	37.0	11.2	44.6	15.4	35.4	6.3
Secondary with specialization	47.3	13.8	42.6	14.3	37.7	8.5
Higher	48.0	11.2	47.3	12.7	38.8	10.9

## Data Availability

The data used to support the findings of this study are available from the corresponding author upon request.
